# Lost in the bloom: DNA-PKcs in green plants

**DOI:** 10.3389/fpls.2023.1231678

**Published:** 2023-07-28

**Authors:** Koppolu Raja Rajesh Kumar

**Affiliations:** Department of Biotechnology, Indira Gandhi National Tribal University, Amarkantak, India

**Keywords:** DNA repair, DNA damage response (DDR), non-homologous end joining (NHEJ), gene loss, plant genome, angiosperms, genome-editing

## Abstract

The DNA-dependent protein kinase catalytic subunit (DNA-PKcs) is a protein encoded by the PRKDC gene in humans and plays a crucial role in repairing DNA double-strand breaks (DSBs). Recent studies have revealed that DNA-PKcs has additional functions in the cell beyond DSB repair, including transcriptional regulation, telomere protection and capping, preserving chromosomal integrity, and regulating senescence, apoptosis, and autophagy. Moreover, DNA-PKcs has also been implicated in regulating the innate immune response, and dysregulation of DNA-PKcs has been commonly observed in various types of cancers. Until recently it was believed that DNA-PKcs is not present in plants in general. However, DNA-PKcs is conserved in green plants ranging from microscopic green algae such as *Ostreococcus* of the chlorophytes to the tallest living trees on earth, *Sequoia* of the gymnosperms. Interestingly, DNA-PKcs has not been detected in angiosperms, or in basal angiosperms which are considered sister groups to all other flowering plants. The long polypeptide and gene length of DNA-PKcs coupled with errors in genome assembly, annotation, and gene prediction, have contributed to the challenges in detecting and extracting DNA-PKcs sequences in plant lineages. Sequence alignment showed that several amino acids throughout the length of DNA-PKcs are conserved between plants and human, and all the typical domains identified in human DNA-PKcs are also found in DNA-PKcs from green plants suggesting possible structural and functional conservation. Given the highly conserved nature of DNA repair pathways between mammals and plants further highlights the potential significance of DNA-PKcs in plant biology.

## 1 Introduction

All organisms experience DNA damage induced by endogenous factors inherent to their cellular activity, as well as exogenous factors present in their environment. Consequently, organisms have evolved mechanisms to effectively address DNA damage and protect the integrity of their genomic information. As sessile organisms, plants are vulnerable to environmental stresses that damage DNA and negatively affect their growth and development. The studies and knowledge on DNA repair mechanisms in plants lags behind mammalian and other model systems such as bacteria and yeast (Manova and Gruszka, 2015). However, the emergence of genome-editing techniques has underscored the critical significance of understanding plant DNA-repair mechanisms. DNA damage especially the DNA double-strand breaks (DSBs) can threaten genomic-integrity, and have the potential to cause cytotoxicity and cell death if left unrepaired (Ceccaldi et al., 2016). Hence the detection and repair of DSBs is essential for survival of all organisms. There are two main repair pathways for DSB repair in both plants and animals: nonhomologous end-joining (NHEJ) and homologous recombination (HR). NHEJ is a homology-independent blunt end joining of DSBs, whereas HR is homology-dependent repair mechanism (Karanam et al., 2012). In plants NHEJ is predominantly works in the repair of DSBs in somatic cells and DSBs induced during meiosis are repaired by HR (Gehrke et al., 2022). Though error-prone, NHEJ plays an important role in maintaining genome-integrity. The initiation of NHEJ occurs when the KU70/80 heterodimer recognizes the ends of the DSBs and recruits the DNA-dependent protein kinase catalytic subunit (DNA-PKcs) to the site of damage. The complex made up of DNA, KU70/80, and DNA-PKcs is known as the DNA-PK or holoenzyme and is essential for the recruitment and regulation of other NHEJ factors such as DNA ligase IV, XRCC4, XLF, Artemis endonuclease, and others that are involved in the processing and ligation of broken DNA ends (Yin et al., 2017).

**Figure 1:**
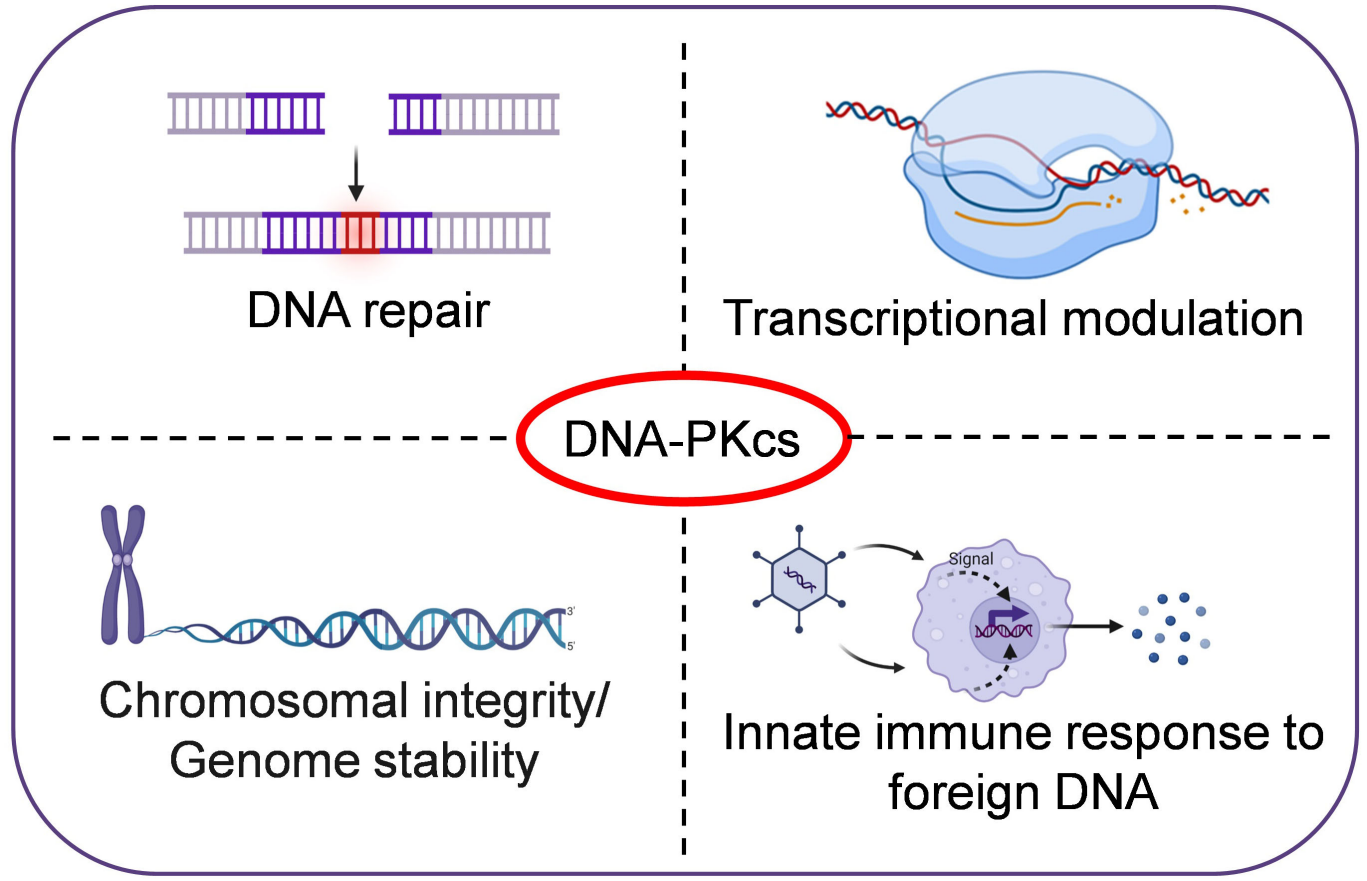
DNA-PKcs, a multifunctional protein in humans. In addition to its role in DNA repair, DNA-PKcs is also involved in transcriptional regulation, maintaining genomic-integrity during mitosis, promoting telomere protection, preserving chromosomal-integrity, and regulating senescence and innate immune responses etc.

DNA-PKcs is a member of the phosphatidyl inositol-3 kinase-like (PIKK) family of protein kinases. The PIKKs share similarities to the eukaryotic protein kinases (ePKs) in terms of catalytic site, but lack the GXGXXG ATP-binding motif found in ePKs (Manning et al., 2002). DNA-PKcs is encoded by the PRKDC gene in humans and is crucial for DNA double-strand break (DSB) repair in mammalian cells. Cell lines deficient in DNA-PKcs from mouse, hamster, and human exhibit defects in DSB repair and V(D)J-recombination, increased sensitivity to ionizing-radiation and drugs such as etoposide and bleomycin, which induce double-stranded breaks in DNA. Further, DNA-PKcs-deficient animals exhibit severe combined immunodeficiencies (SCID) phenotype (Matsumoto et al., 2021). However, recent studies have shown that DNA-PKcs also has other functions in the cell, such as transcriptional regulation, and phosphorylating targets outside the DNA damage response (**Figure 1**). Through its non-nuclear functions in cells, DNA-PKcs is involved in maintaining genomic-integrity during mitosis, regulating metabolism, promoting telomere protection, preserving chromosomal-integrity and regulating senescence, apoptosis, autophagy and the innate immune response. Dysregulation of DNA-PKcs is commonly found in various types of cancers (Bartlett and Lees-Miller, 2018; Dylgjeri and Knudsen, 2022).

Green plants, also known as Viridiplantae, are a monophyletic group of eukaryotic organisms that includes more than 450,000 species (Lughadha et al., 2016; One-Thousand-Plant-Transcriptomes-Initiative, 2019). Viridiplantae are classified into two sister groups: Chlorophyta (green-algae) and Streptophyta. The Streptophyta group encompasses charophyte algae and all embryophytes, including bryophytes and vascular plants. Charophytes or streptophyte algae are considered to be the ancestors of modern-day land plants. During the early colonization of land by plants, a diversification event occurred resulting in the emergence of three distinct lineages that were gametophyte-dominant, collectively known as bryophytes (hornworts, liverworts, and mosses). Additionally, a fourth lineage with a dominant sporophyte generation, the tracheophytes or vascular plants, also evolved during this period (Sousa et al., 2020). Within vascular plants, lycophytes are sisters to the remaining land plants or Euphyllophyta, which comprise Monilophytes (ferns and fern-allies) and spermatophytes (seed plants). Spermatophytes include two major groups: gymnosperms and angiosperms.

Although DNA-PKcs has multiple functions in mammals and new functions are continuously being uncovered for this enigmatic protein, its status in plants remains unclear. The availability of an increasing number of plant genomes from various lineages has revealed that DNA-PKcs is present in some plant groups (Lees-Miller et al., 2021). Nevertheless, a comprehensive analysis of DNA-PKcs across different plant lineages is currently lacking. To clarify this, I conducted a search for DNA-PKcs homologs in various lineages of green plants. Further, I attempted to obtain and analyze the full-length coding sequences, protein sequences, and genomic sequences of DNA-PKcs from different plant lineages based on the available genome and transcriptome data from each lineage.

## 2 Methods

### 2.1 Searching the plant kingdom for DNA-PKcs homologs

To determine the presence of DNA-PKcs in various plant lineages, I utilized the human DNA-PKcs protein sequence as a query and conducted BLASTP and TBLASTN searches against publicly available databases, including NCBI, Phytozome (https://phytozome-next.jgi.doe.gov/), and the 1KP databases (One-Thousand-Plant-Transcriptomes-Initiative, 2019). The searches were performed on the Genome, WGS, EST, and transcriptome shotgun assembly (TSA) databases. Afterwards, plant DNA-PKcs protein sequences, such as those obtained from *Pinus*, were used once again as a query to conduct additional searches on the aforementioned databases. To prevent matching with other PIKK members that share kinase and FATC domains, the N-terminal part of DNA-PKcs was used exclusively, and the kinase and FATC domains were excluded from the query sequence. Apart from transcriptome and whole-genome shotgun contigs (WGS) searches, over thirty complete genomes from dicots and monocots were also examined for DNA-PKcs using TBLASTN. Independent genomes of various green plants lineages were analyzed at NCBI Genome (https://www.ncbi.nlm.nih.gov/genome/), Phytozome (https://phytozome-next.jgi.doe.gov/), and Phycocosm (https://phycocosm.jgi.doe.gov/).

### 2.2 Obtaining putative full-length DNA-PKcs sequences

To obtain putative DNA-PKcs proteins, their respective CDS and genomic sequences, the genomes of various plant species belonging to eight different lineages were analyzed. This involved selecting existing annotations in some species, generating new annotations using available transcript information, and predicting genes based on DNA-PKcs proteins with transcript support in some other species or using available TSA directly for predicting protein sequence (**Supplementary Table 1**).

Briefly, the existing annotation of DNA-PKcs in *Bathycoccus prasinos* and *Ostreococcus tauri* were used. In the *Klebsormidium nitens* genome (formerly known as *Klebsormidium flaccidum*), DNA-PKcs was identified on scaffold DF237153, but was annotated as two separate proteins (GAQ84758.1 and GAQ84757.1). To obtain the full-length protein, TSA available at NCBI (GBSO01008411.1), which spans both predicted proteins, was utilized. Additionally, the N-terminal part of the protein was obtained from another TSA (GBSO01005714.1). The predicted *Mesotaenium endlicherianum* protein sequence that matches DNA-PKcs is 4624 aa long and lacks the FAT domain (ME000610S08690). It may potentially be longer by almost 500 aa compared to the typical DNA-PKcs protein. To predict the DNA-PKcs gene in *Mesotaenium endlicherianum*, GeneWise (Birney et al., 2004), a homology-based gene predictor, was utilized with the *Anthoceros agrestis* DNA-PKcs protein as reference protein. To predict the coding sequence (CDS) and protein of *Anthoceros agrestis* DNA-PKcs, I downloaded the TSA sequence encoding the full-length protein from the 1KP database. This TSA sequence was used as a reference to predict both the CDS and protein sequences using bioinformatic tools. The *Azolla filiculoides* DNA-PKcs sequence predicted at fernbase.org is incomplete (Azfi_s0095.g043658). Hence, the sequence of Azolla DNA-PKcs was predicted using GeneWise with *Ceratopteris richardii* protein as a reference. To obtain putative DNA-PKcs gene and protein sequences for *Ceratopteris richardii* and *Sequoia sempervirens*, I downloaded the respective TSA sequence that harbor full-length DNA-PKcs from the NCBI database. However, in both cases, part of the DNA-PKcs sequence present in the TSA is missing from the available genome scaffolds, which prevented me from obtaining the full-length gene sequence. The CDS and protein sequences of DNA-PKcs from *Pinus taeda* were predicted using the TSA support from *Pinus flexilis.* Corresponding *Pinus taeda* gene sequence was obtained from Spruce Genome Project (congenie.org).

To obtain the DNA-PKcs gene and protein sequences for *Marchantia polymorpha*, *Sphagnum fallax* and *Diphasiastrum complanatum*, existing annotations available at phytozome were used. Similarly, for *Physcomitrella patens* and *Selaginella moellendorffii* existing annotations available at NCBI were used (**Supplementary Table 1**). The selection of an annotation or gene-prediction for analysis was based on the presence of all the typical domains (N-HEAT, M-HEAT, FAT, KINASE and FATC) of DNA-PKcs.

### 2.3 Multiple sequence alignment and phylogenetic analysis

Multiple sequence alignment of putative DNA-PKcs proteins from green plants was performed using COBALT (Papadopoulos and Agarwala, 2007) and Clustal Omega (Sievers and Higgins, 2018) using default parameters. In COBALT the regions of high amino acid similarity/conservation are represented by red, regions of moderate conservation in blue, and non-conserved amino acids in grey. The alignment generated using Clustal Omega was visualized using GeneDoc. The amino acid conservation in the DNA-PKcs protein sequences is indicated by shading, with black, dark grey, and light grey representing 100%, 80%, and 60% conservation, respectively. Conservative substitutions are considered identical for the purpose of shading. Phylogenetic tree was generated online at https://ngphylogeny.fr/. Clustal Omega was employed for multiple sequence alignment, and BMGE (Block Mapping and Gathering with Entropy) was utilized to curate the alignment. PhyML was used for the phylogenetic tree interface. The phylogenetic tree was visualized using Interactive Tree of Life (Lemoine et al., 2019; Letunic and Bork, 2021).

### 2.4 Conserved domain detection, gene structure analysis and subcellular location prediction

The putative DNA-PKcs protein sequences were scanned using hmmscan and InterPro (available at www.ebi.ac.uk) to detect conserved Pfam-domains. Gene-Structure-Display-Server (GSDS) (Hu et al., 2015) was utilized to analyze and display the putative gene structure of DNA-PKcs in different plant species. To predict the protein subcellular localization, LOCALIZER tool available at https://localizer.csiro.au/ was used.

## 3 Results and discussion

**Figure 2:**
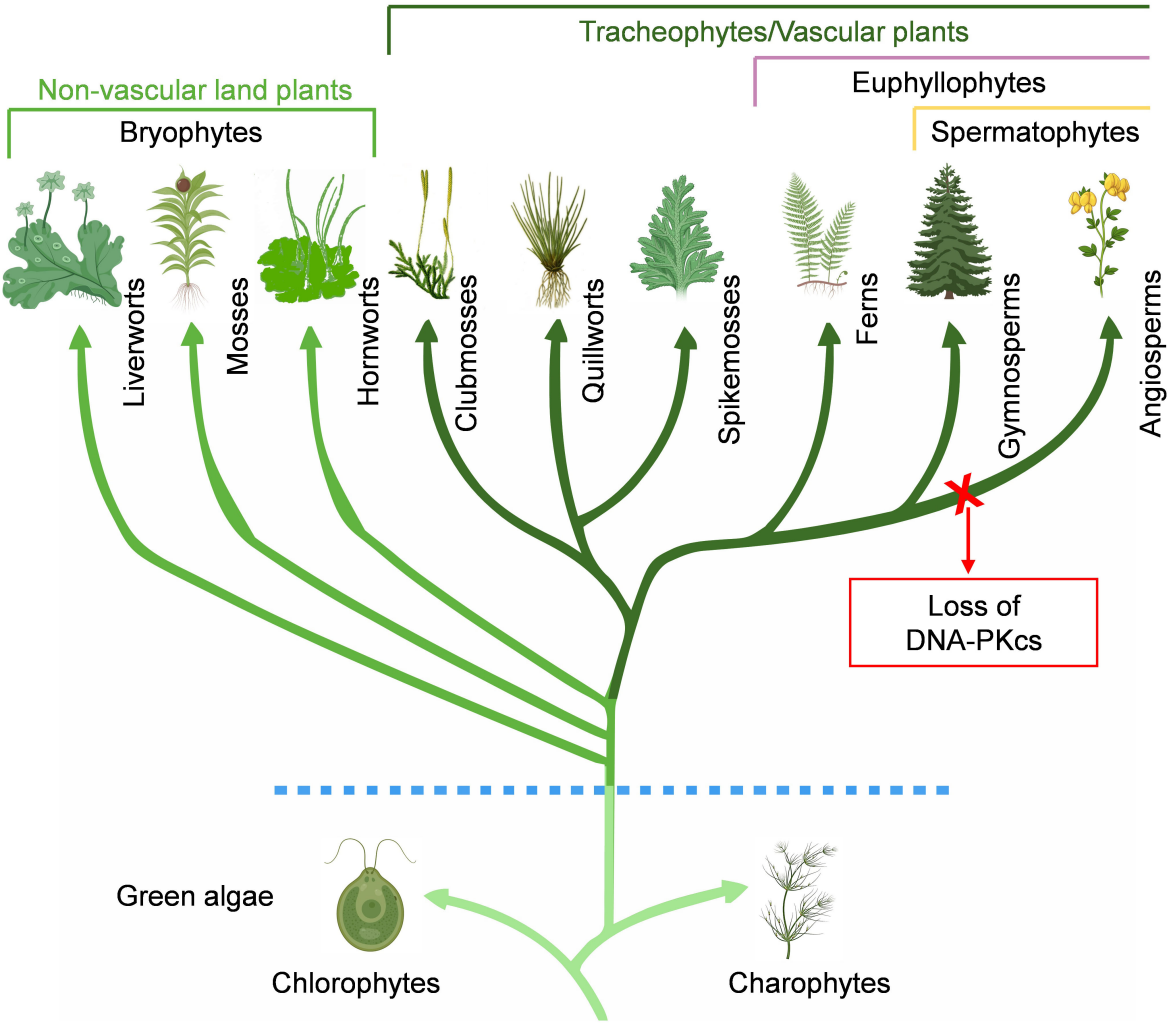
Conservation of DNA-PKcs in Viridiplantae. DNA-PKcs homologs are found in chlorophytes, charophytes, bryophytes (liverworts, mosses, hornworts), lycophytes (clubmosses, quillworts, spikemosses), monilophytes, and gymnosperms. Notably, DNA-PKcs homologs are not found in basal angiosperms, monocots and dicots, and appears to have been lost during the divergence of angiosperms.

The search for DNA-PKcs in plant kingdom revealed the presence of DNA-PKcs homologs in chlorophytes, charophytes, different lineages of bryophytes (liverworts, mosses, hornworts), lycophytes (clubmosses, quillworts, spikemosses), monilophytes, and gymnosperms (**Figure 2**). In gymnosperms there are four extant lineages: conifers, cycads, ginkgoes, and gnetophytes. Among them, DNA-PKcs is found in both conifers and cycads, as well as in the only representative of ginkgoes, *Ginkgo biloba*. Gnetophytes comprise three genera, *Gnetum*, *Welwitschia*, and *Ephedra*, which are morphologically distinct from other seed plants. Notably, DNA-PKcs is found in all three genera of gnetophytes. The Spermatophyta group of vascular plants is incredibly diverse group consisting of over 350,000 species, with angiosperms being the main contributor to this diversity (Lughadha et al., 2016). However, DNA-PKcs has not been detected in the monocots and eudicots, which make up the majority of living angiosperms, or in other small groups like chloranthales, magnoliids, and ceratophyllales. Additionally, DNA-PKcs was not found in basal angiosperms such as amborellales, nymphaeales, and austrobaileyales, collectively known as ANA grade, which are basal groups of angiosperms and considered sister group to all other flowering plants. Based on this, it is plausible that DNA-PKcs was conserved during much of the evolutionary history of green plants and was present in the common ancestor of seed plants. However, it appears to have been lost during the divergence of angiosperms (**Figure 2**). The mechanisms and components of DNA-repair in plants were mostly elucidated from Arabidopsis, an angiosperm and the first plant to have its genome sequenced (Hays, 2002). This, coupled with the fact that other early sequenced plant genomes also belonged to angiosperms, led to the assumption until recently that DNA-PKcs is not present in plants in general (Nishizawa-Yokoi et al., 2012; Schmidt et al., 2019). The search for DNA-PKcs in TSA databases yielded some hits in the angiosperms. However, upon closer inspection, these were found to originate from fungi and aphids, indicating contamination in the plant transcriptomes.

Although searching for the presence of DNA-PKcs in different plant lineages is relatively straightforward with available genome and transcriptome data, obtaining putative full-length protein and gene sequences is challenging due to the very long polypeptide (Human DNA-PKcs: 4128 amino acids) and corresponding CDS/gene length. This is compounded by errors in genome assembly, annotation, and gene prediction, as well as a lack of sufficient transcript information and poor sequence quality in some genomes. For example, partial protein sequences of DNA-PKcs are often predicted and annotated as two separate proteins, each carrying different domains of DNA-PKcs. This can be observed in the genomes of *Chlamydomonas eustigma*, *Coccomyxa subellipsoidea*, *Klebsormidium nitens*, and *Isoetes taiwanensis*, the representative genome of quillworts (**Supplementary Table 1**). Further, in the genome of *Mesotaenium endlicherianum*, DNA-PKcs is predicted to be a polypeptide of 4624 amino acids. However, the FAT domain of DNA-PKcs is not present in the predicted protein or CDS, despite being encoded in the gene sequence suggesting potential gene prediction-errors. DNA-PKcs is annotated as a hypothetical protein (of 3531 aa) in the *Chara braunii* genome (**Supplementary Table 1**). The N-terminal part of this protein contains long stretches of repetitive amino acids (~250 aa), suggesting low quality of the *Chara braunii* genome sequence at least in the region of this gene/scaffold under investigation. In addition to this, a partial TSA sequence of *Chara braunii* (GGXX01117481.1) encoding a conserved part of DNA-PKcs domain (DNAPKcs_CC5 domain, Pfam19704) was not found in the predicted CDS and protein sequence of *Chara braunii* DNA-PKcs and was even missing from the corresponding genome scaffold (BFEA01000767.1, scaffold_798) harboring the gene, ruling out any gene prediction-error. Further analysis revealed that this particular transcript sequence is present in another scaffold (BFEA01001808.1, scaffold_3591), indicating a possible assembling-error. These examples demonstrate the need for greater transcript sequence information, in order to predict DNA-PKcs with higher confidence and to facilitate successful genome assembly and gene prediction in general.

A total of 14 putative DNA-PKcs proteins, along with their respective CDS and genomic sequences, were extracted from the genomes of various plant species representing eight different lineages for further analysis. This was accomplished by selecting existing annotations in some species, generating new annotations using available transcript information, and predicting genes based on DNA-PKcs proteins with transcript support in some other species or using available TSA directly for predicting protein sequence (**Supplementary Table 1**). Overall. protein sequences were predicted using various methods in an effort to obtain at least one to two candidates of DNA-PKcs from each plant lineage. But because of the unusually long length of the protein, errors are likely to occur in some of these predicted proteins. The extracted DNA-PKcs proteins are from *Bathycoccus prasinos* and *Ostreococcus tauri* of chlorophytes, *Mesotaenium endlicherianum* and *Klebsormidium nitens* of charophytes, *Marchantia polymorpha* of liverworts, *Anthoceros agrestis* of hornworts, *Physcomitrella patens* and *Sphagnum fallax* of mosses, *Diphasiastrum complanatum* and *Selaginella moellendorffii* of lycophytes, *Ceratopteris richardii* and *Azolla filiculoides* of monilophytes, *Sequoia sempervirens* and *Pinus taeda* of gymnosperms.

**Figure 3:**
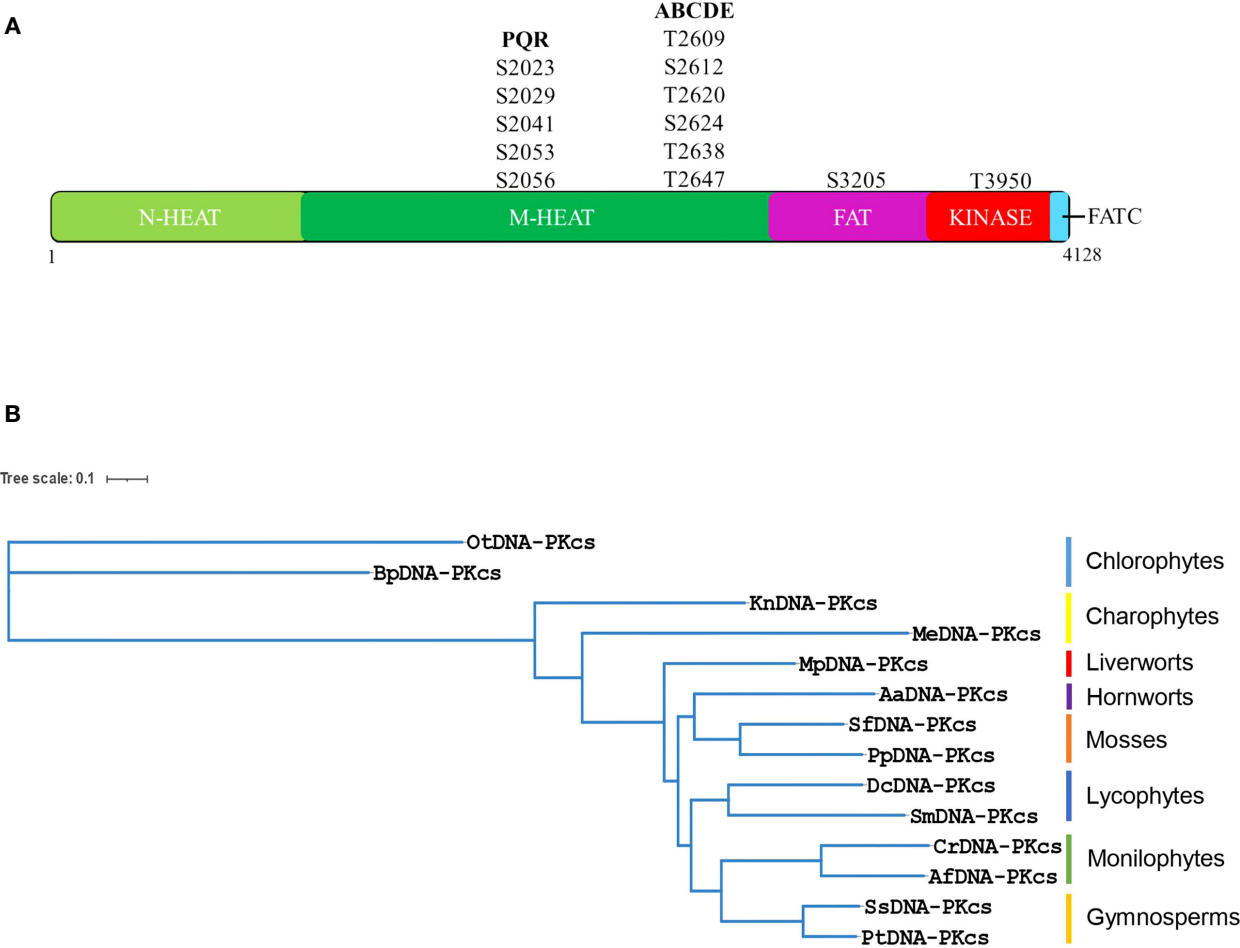
**(A)** Human DNA-PKcs structure showing major domains and important autophosphorylation sites including PQR and ABCDE clusters. DNA-PKcs is composed of a N-terminal HEAT domain (N-HEAT), a middle-HEAT domain, also known as circular-cradle (M-HEAT), the FAT domain the kinase domain and the FATC domain. **(B)** Phylogenetic tree of putative DNA-PKcs protein homologs from representative species of different lineages of green plants. The phylogenetic tree was constructed using the maximum likelihood method using the online platform NGPhylogeny. (Bp, *Bathycoccus prasinos*; Ot, *Ostreococcus tauri*; Me, *Mesotaenium endlicherianum*; Kn, *Klebsormidium nitens*; Mp, *Marchantia polymorpha*; Pp, *Physcomitrella patens*; Sf, *Sphagnum fallax*; Aa, *Anthoceros agrestis*; Dc, *Diphasiastrum complanatum*; Sm, *Selaginella moellendorffii*; Cr, *Ceratopteris richardii*; Af, *Azolla filiculoides*; Ss, *Sequoia sempervirens*; Pt, *Pinus taeda)*.

The DNA-PKcs protein is composed of various domains, including a large N-terminal HEAT-repeat containing domain, a conserved FAT (FRAP, ATM and TRRAP) domain, a kinase-domain and a short C-terminal FATC-domain (**Figure 3A**). The N-terminal region is further divided into an N-terminal HEAT-repeat domain (N-HEAT) and a middle (M-HEAT) domain that forms an alpha-solenoid, also known as the circular-cradle (Sibanda et al., 2017). DNA-PKcs also contains several important phosphorylation sites, including the ABCDE and PQR clusters. Multiple sequence alignment of all the selected plant DNA-PKcs proteins along with human DNA-PKcs showed that several amino acids at the entire length of DNA-PKcs showed conservation between plant and human DNA-PKcs (**Supplementary Figures 1**, **2**). And a greater extent of conservation was observed among the DNA-PKcs homologs of plants (**Supplementary Figure 2**). In particular, the region spanning the end of the N-terminal HEAT-repeat domain (N-HEAT) and the starting of the middle (M-HEAT) domain (Pfam domain: DNAPKcs_CC1/2), as well as the DNAPKcs_CC5 domain, FAT-domain, kinase domain, and FATC-domain regions of DNA-PKcs exhibited higher conservation between plant DNA-PKcs homologs and human DNA-PKcs (**Supplementary Figures 1**, **2**). Moreover, all the typical Pfam domains identified in human DNA-PKcs were also found in DNA-PKcs from green plants (**Supplementary Figure 3**). A recently reported YRPD-motif (YR-x-G-(D/E)-(L/F)-PD-(I/V)-x-I-x5-I-x-P-x-Q) starting at tyrosine 2775 of human DNA-PKcs is found to be conserved in metazoans, and the residues YRxGxxPD in particular are conserved across all organisms analyzed by Lees-Miller et al. (2021), indicating critical role for YRPD-motif in DNA-PKcs function. Analysis of plant DNA-PKcs proteins revealed that the YRPD-motif is also conserved in green plants (YR-x-G-(D/E)-(L/Y)-PD-(I/V)-x-I-x5-(I/V/L)-x-(P/L)-x-x) with slight differences compared to metazoans. The glutamine (Q) at position 2795 in the human YRPD domain is not conserved and is completely absent in all green plants studied. Moreover, the second YR sequence at tyrosine 2772 and arginine 2773, which is conserved in metazoans, is modified in plants, with arginine (R) being conserved in all plants analyzed and tyrosine (Y) being replaced by methionine (M), leucine (L), threonine (T), and valine (V) (**Supplementary Figure 4**).

DNA-PKcs contains several important phosphorylation and autophosphorylation sites. Two major regions of autophosphorylation on the human DNA-PKcs protein are referred to as PQR and ABCDE clusters. The PQR cluster contains five serine sites (serines 2023, 2029, 2041, 2053, and 2056), while the ABCDE cluster contains six sites of serine/threonine (T2609, S2612, T2620, S2624, T2638, and T2647). Phosphorylation at these sites plays important roles in DSB-repair and V(D)J-recombination. However, upon analysis of plant DNA-PKcs, it was found that none of the five serines corresponding to the PQR cluster of human DNA-PKcs are conserved in green plants (**Supplementary Figure 5**). Nevertheless, several serines are present in the vicinity of the PQR cluster in green plants, and it is possible that these serine residues function in autophosphorylation events and act as DNA-PKcs-dependent phosphorylation sites in plants. Within the ABCDE cluster, threonine 2609 is conserved in most of the plants analyzed, suggesting a possible functional role in these organisms (**Supplementary Figure 6**). However, in *Marchantia* and *Physcomitrella*, it is substituted by serine, in *Selaginella* it is replaced by alanine, and in *Ostreococcus* and *Bathycoccus*, it is replaced by glutamic acid and methionine, respectively. S2612, T2620, and S2624 are not conserved in green plants. The T2638 site is invariant in all plants analyzed, indicating its importance for DNA-PKcs function across different species, while T2647 is substituted with serine in most plants (**Supplementary Figure 6**). Other autophosphorylation sites of DNA-PKcs have been identified *in vitro*, such as S3205 in FAT domain and T3950 in the kinase domain (Douglas et al., 2002). S3205 is not conserved in green plants, whereas T3950 is conserved in the analyzed chlorophytes and charophytes but exhibits substitutions by valine and isoleucine in other plant lineages (**Supplementary Figures 7**, **8**). Although these autophosphorylation sites are important, they represent only a small portion of the total post-translational modifications identified on human DNA-PKcs. In fact, around 88 phosphoserines, 34 phosphothreonines, and 21 phosphotyrosines have been identified on this protein (Lees-Miller et al., 2021).

To gain insight into the evolutionary relationships of putative DNA-PKcs proteins, a phylogenetic tree was constructed using homologous proteins from 14 species of green plants across eight different lineages. Chlorophytes, which make up the majority of green algal diversity, are relatively distantly related to land plants, while charophytes are considered to be close relatives of land plants (Delwiche and Cooper, 2015). In this study, two charophyte species were examined: *Klebsormidium nitens*, which belongs to the Klebsormidiophyceae, and *Mesotaenium endlicherianum*, which belongs to the Zygnematophyceae. Phylogenetic analysis of putative DNA-PKcs proteins indicated that *Mesotaenium endlicherianum* is closer to bryophytes, the earliest-diverging land plants, than *Klebsormidium nitens* or chlorophytes. Therefore, the evolution of DNA-PKcs is consistent with the previously determined phylogenetic positioning of Klebsormidiophyceae and Zygnematophyceae members of charophytes in relation to land plants (**Figure 3B**) (Delwiche and Cooper, 2015), and is also consistent with the phylogenetic positioning of other lineages of green plants (**Figure 3B**). The bioinformatic analysis of putative plant DNA-PKcs proteins revealed a predicted molecular weight in the range of around 460-470 kDa, similar to that of human DNA-PKcs. Additionally, all plant DNA-PKcs proteins were predicted to localize to the nucleus (**Supplementary Figure 9**). Further analysis of putative gene structure showed that DNA-PKcs in plant exhibited diverse gene lengths owing to the different intron length in different species and lineages (**Supplementary Figure 10**). The DNA-PKcs gene in *Bathycoccus prasinos* lacks introns, which is interesting when compared to other chlorophytes such as *Chlamydomonas*, *Volvox*, and *Coccomyxa*, all of which contain introns in their respective DNA-PKcs genes. Gymnosperms are known to harbor some of the longest introns in the plant kingdom, with putative DNA-PKcs gene lengths of approximately 107 kb in *Gnetum montanum*, ~306 kb in *Taxus wallichiana*, ~504 kb in *Pinus taeda* and ~794 kb in *Cycas panzhihuaensis*.

Although the DNA-PKcs protein plays critical roles in mammals, it is absent from several species. It is conserved in species such as humans, mouse, chicken, and mosquito, but not found in model organisms like *Drosophila melanogaster*, *Caenorhabditis elegans*, and *Saccharomyces cerevisiae* (Bartlett and Lees-Miller, 2018). Although DNA-PKcs is conserved in the plant kingdom from chlorophytes to gymnosperms, it is also possible that it has been lost independently by some species. For instance, while DNA-PKcs is not found in *Chlamydomonas reinhardii*, it is present in *Chlamydomonas eustigma*. Given the complex evolutionary history of DNA-PKcs, it is not surprising that it is not found in certain species, although the functional relevance of this remains to be understood. The DNA-PKcs has not received the attention in plant biology partly due to its absence in angiosperms, which include crop plants. Nonetheless, given its critical roles reported in animal systems and humans, it is reasonable to assume that it also plays a critical role in plants.

As discussed earlier, in some plant genomes though DNA-PKcs was present, it was not annotated or included in the predicted encoded proteins, or sometimes annotated as two separate proteins. These examples illustrate that the assembly and annotation of genomes can be complex and challenging particularly with a long and uncharacterized gene like DNA-PKcs which in not present in angiosperm model plants. Although DNA-PKcs appears to be lost in angiosperms, other PIKK members involved in DNA damage response, such as ATM and ATR, are present and activated in DDR signaling (Culligan et al., 2006). DNA-PKcs and ATM share similarities in their primary structure and are both implicated in cellular responses to DNA double-stranded breaks. While ATM plays a more prominent role in the cell cycle checkpoint, there are significant overlaps in the functions and protein phosphorylation between DNA-PKcs and ATM. Both proteins undergo complex regulation through autophosphorylation and mutual phosphorylation (Matsumoto et al., 2021). It is possible that some of the PIKK family members may have evolved additional functions to compensate the loss of DNA-PKcs in angiosperms.

The current understanding of the functional significance of DNA-PKcs is primarily from studies on human and mouse DNA-PKcs. Due to the absence of DNA-PKcs in other important model organisms like yeast and drosophila, it is not clear if the DNA-PKcs in humans has gained any unique functions or if DNA-PKcs shows functional conservation across different species, including plants. Nonetheless, the highly conserved nature of DNA-repair pathways between mammals and plants (Gehrke et al., 2022) further highlights the potential significance of DNA-PKcs in plant biology.

The absence of DNA-PKcs in angiosperms suggests that the NHEJ mechanism and DNA-repair mechanism in general may not be entirely conserved in all plant lineages, and may vary between angiosperms and other plant lineages. Based on the current analysis, it is worth noting that DNA-PKcs is not only lost in angiosperm evolution but can also get lost during the assembly and annotation of plant genome sequences. This highlights the need for better gene prediction and annotation tools for different plant lineages. Genome-editing is becoming increasingly promising for precision engineering of crops and can become a powerful tool for future food security. The generation of long tracts of 3’ssDNA overhangs at DSBs through extensive DNA end resection is a crucial step that commits the cell to utilize HR for repairing DSBs. Recent study using a genome-wide CRISPR/Cas9 screen found that DNA-PKcs as a component of the DNA-PK complex, plays a critical role in promoting DNA end resection in mammalian G0 cells. Further, the kinase activity of DNA-PK was found to be critical, as resection of DSBs diminished upon DNA-PK inhibitor treatment (Fowler et al. 2022). Hence, investigating the function of DNA-PKcs in plants would not only shed light on their DNA-repair mechanism and the evolution of repair mechanisms, but it could also open new avenues for optimizing the efficiency of manipulating plant genomes. Since DNA-repair is pivotal for all genetic engineering capabilities, including genome-editing, understanding DNA-PKcs in plants could likely expand the toolbox for genome engineering in plants even further.

## Data availability statement

The original contributions presented in the study are included in the article/**Supplementary Material**. Further inquiries can be directed to the corresponding author.

## Author contributions

KRRK: Conceptualization, bioinformatic analysis, manuscript writing.

## Supplementary material

Supplementary Table 1 Details of putative DNA-PKcs gene/protein annotations used or discussed in the current study from different plant species.

Supplementary Figure 2 Multiple sequence alignment of putative DNA-PKcs from various plant lineages and Human DNA-PKcs. The alignment was generated using Clustal Omega and visualized using GeneDoc. The amino acid conservation in the DNA-PKcs protein sequences is indicated by shading, with black, dark grey, and light grey representing 100%, 80%, and 60% conservation, respectively. It is important to note that conservative substitutions are considered identical for the purpose of shading. The putative DNA-PKcs sequences are from Bp (*Bathycoccus prasinos*), Ot (*Ostreococcus tauri*), Me (*Mesotaenium endlicherianum*), Kn (*Klebsormidium nitens*), Mp (*Marchantia polymorpha*), Pp (*Physcomitrella patens*), Sf (*Sphagnum fallax*), Aa (*Anthoceros agrestis*), Dc (*Diphasiastrum complanatum*), Sm (*Selaginella moellendorffii*), Cr (*Ceratopteris richardii*), Af (*Azolla filiculoides*), Ss (*Sequoia sempervirens*), Pt (*Pinus taeda*), and DNA-PKcs from Hs (*Homo sapiens*).
