# Supplementary material for: Lost in the bloom: DNA-PKcs in green plants
Source: Front Plant Sci. 2023 Jul 28;14:1231678. doi: 10.3389/fpls.2023.1231678 (PMC10419180; doi:10.3389/fpls.2023.1231678)
Supplement: Supplementary Table 1 — Details of putative DNA-PKcs gene/protein annotations used or discussed in the current study from different plant species. [file DataSheet_1.zip › Supplementary Figures 1-10.PDF]

Supplementary figure 1.

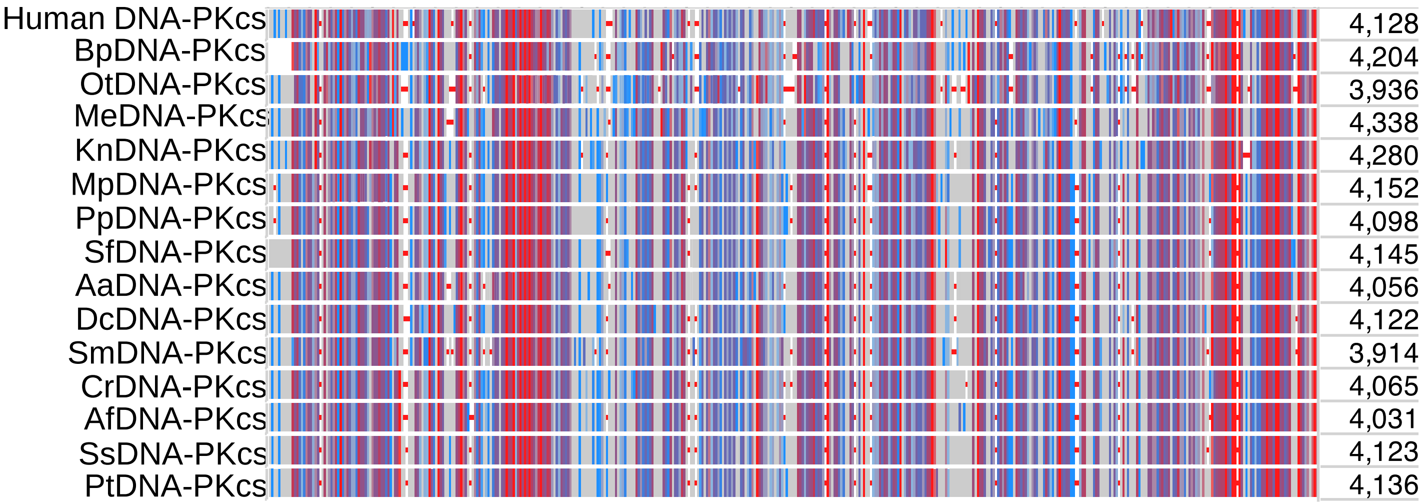

Supplementary figure 1.

Overall view of amino acid conservation in DNA-PKcs proteins of green plants. Putative DNA-PKcs proteins sequences from green plants and DNA-PKcs from human were aligned using COBALT, and regions of high amino acid similarity/conservation are represented by red, regions of moderate conservation in blue, and non-conserved amino acids in grey. The putative DNA-PKcs sequences are from Bp (*Bathycoccus prasinos*), Ot (*Ostreococcus tauri*), Me (*Mesotaenium endlicherianum*), Kn (*Klebsormidium nitens*), Mp (*Marchantia polymorpha*), Pp (*Physcomitrella patens*), Sf (*Sphagnum fallax*), Aa (*Anthoceros agrestis*), Dc (*Diphasiastrum complanatum*), Sm (*Selaginella moellendorffii*), Cr (*Ceratopteris richardii*), Af (*Azolla filiculoides*), Ss (*Sequoia sempervirens*), Pt (*Pinus taeda*), and Hs (*Homo sapiens*).

Supplementary Figure 2.

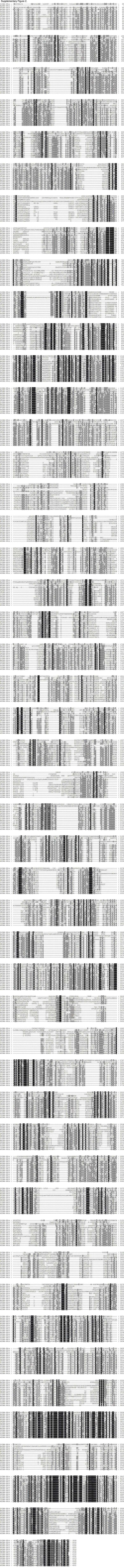

Supplementary figure 3

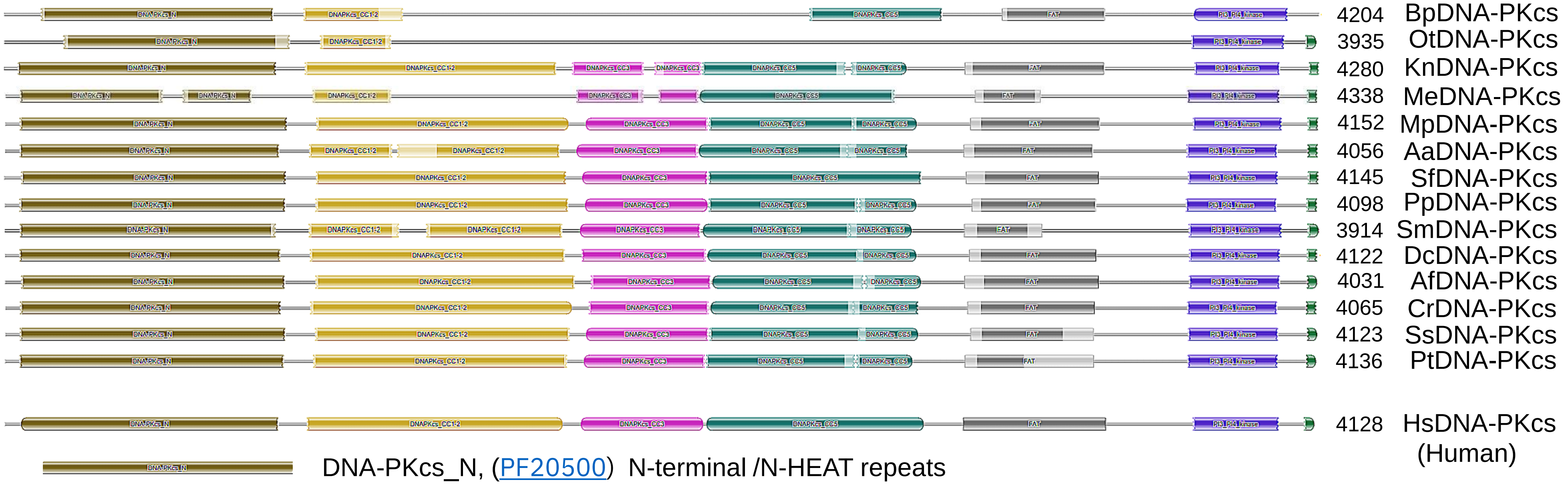

DNA-PKcs_N, (PF20500) N-terminal /N-HEAT repeats

DNAPKcs_CC1-2 (PF20502) (Circular Cradle 1-2)

DNAPKcs_CC3 (PF08163) (This domain contains the Ku-binding site A and a the highly conserved region (HCR) II)

DNAPKcs_CC5 (PF19704) (C-terminal region of the circular cradle segment (CC, also known as M-HEAT repeats)

FAT (PF02259)

PI3_PI4_Kinase (Phosphatidylinositol 3- and 4-kinase) (PF00454)

FATC (PF02260)

Supplementary figure 3

Detection of Pfam domains in human and plant DNA-PKcs. Protein sequence was searched against Pfam database using the program hmmscan. Conserved Pfam domains in human DNA-PKcs were found to be present in the majority of plant proteins, indicating possible conservation of structure and function. Sequence variability below the detection threshold could be the reason for absence of few domains in DNA-PKcs from chlorophytes. The putative DNA-PKcs sequences are from Bp (*Bathycoccus prasinos*), Ot (*Ostreococcus tauri*), Me (*Mesotaenium endlicherianum*), Kn (*Klebsormidium nitens*), Mp (*Marchantia polymorpha*), Pp (*Physcomitrella patens*), Sf (*Sphagnum fallax*), Aa (*Anthoceros agrestis*), Dc (*Diphasiastrum complanatum*), Sm (*Selaginella moellendorffii*), Cr (*Ceratopteris richardii*), Af (*Azolla filiculoides*), Ss (*Sequoia sempervirens*), Pt (*Pinus taeda*), and DNA-PKcs from Hs (*Homo sapiens*).

Supplementary Figure 4.

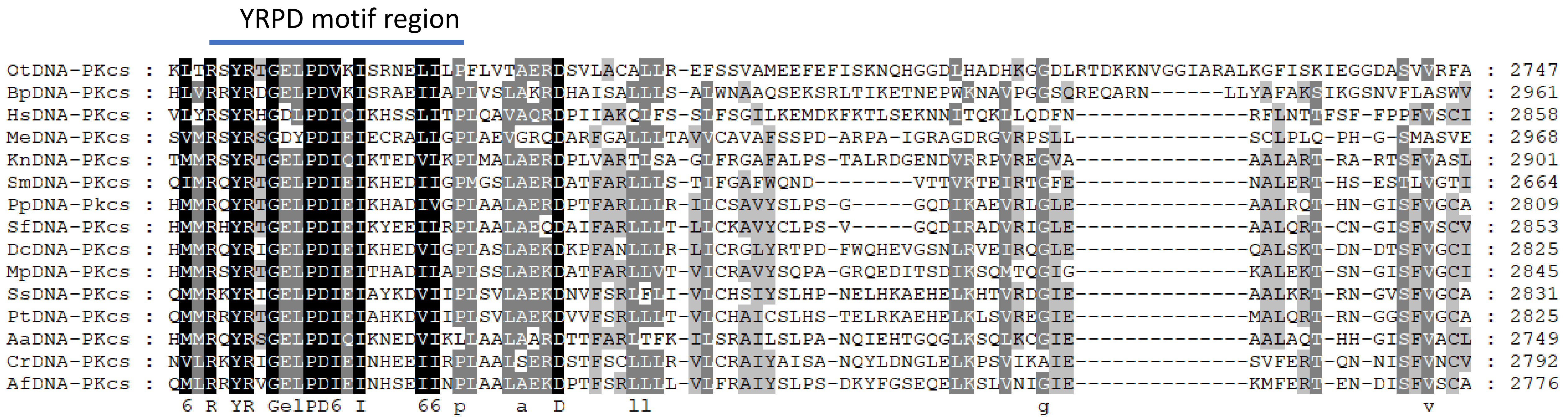

Supplementary Figure 4. Conservation of YRPD motif in green plants. Compared to metazoans, the YRPD motif is slightly modified in green plants (YR-x-G-(D/E)-(L/Y)-PD-(I/V)-x-I-x5-(I/V/L)-x-(P/L)-x-x), however the residues YRxGxxPD are invariant like in other organisms.

Bp- *Bathycoccus prasinos*; Ot- *Ostreococcus tauri*; Me- *Mesotaenium endlicherianum*; Kn- *Klebsormidium nitens*; Mp- *Marchantia polymorpha*; Pp- *Physcomitrella patens*; Sf- *Sphagnum fallax*; Aa- *Anthoceros agrestis*; Dc- *Diphasiastrum complanatum*; Sm- *Selaginella moellendorffii*; Cr- *Ceratopteris richardii*; Af- *Azolla filiculoides*; Ss- *Sequoia sempervirens*; Pt- *Pinus taeda*; Hs-*Homo sapiens*.

Supplementary Figure 5.

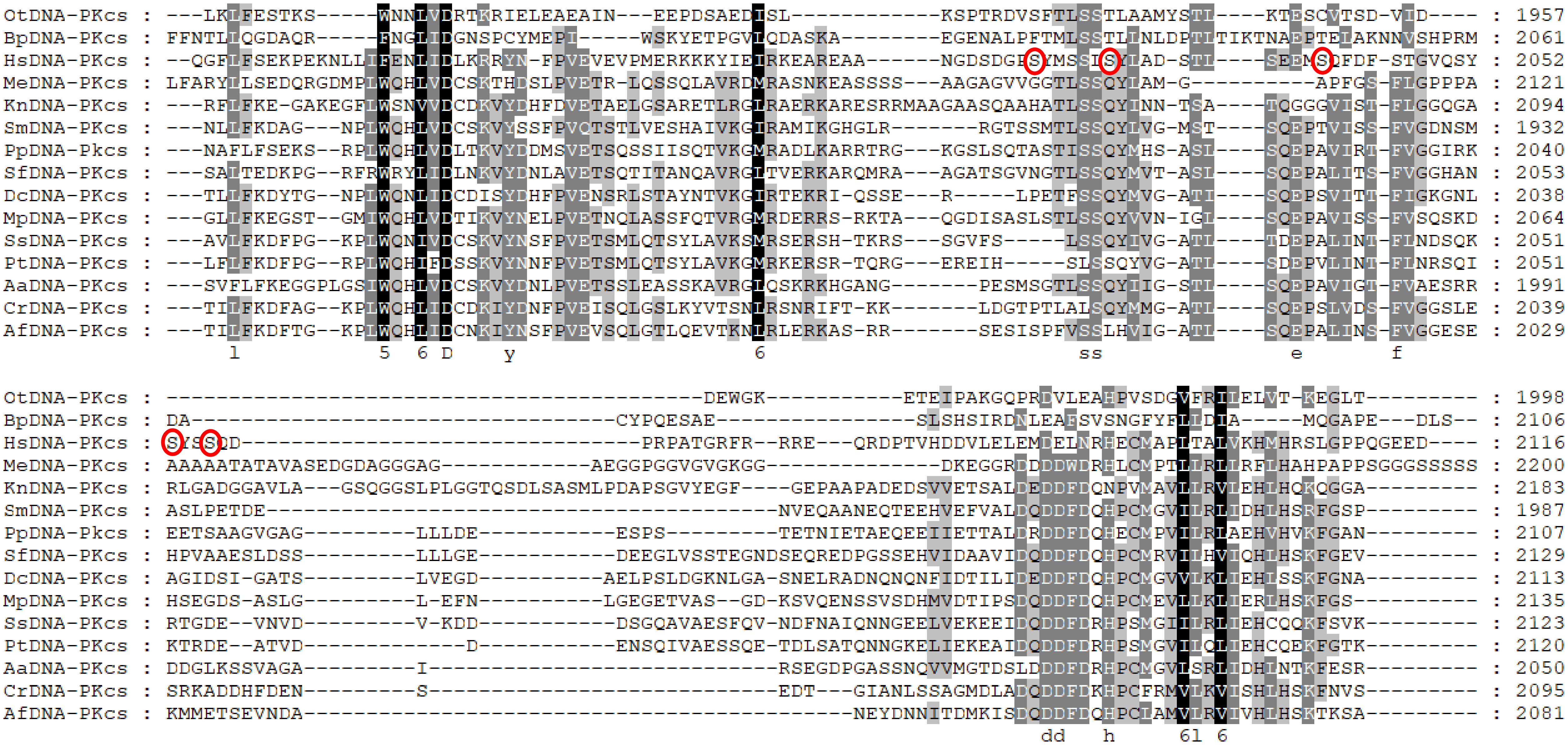

Supplementary Figure 5. PQR cluster of DNA-PKcs. Five serine residues of the PQR cluster of Human DNA-PKcs are marked with red circles. Lack of conservation of the PQR cluster residues in green plants can be observed.

Supplementary Figure 6.

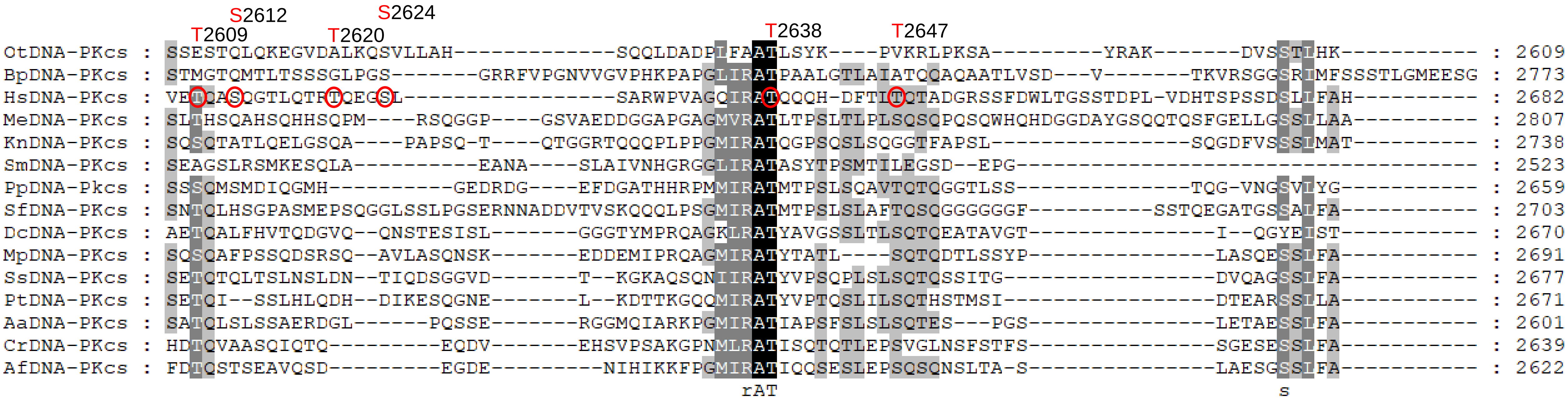

Supplementary Figure 6. ABCDE cluster of DNA-PKcs. The ABCDE cluster is a region of the human DNA-PKcs protein that contains six serine/threonine phosphorylation sites important for its activity. In this figure, these sites are highlighted in red circles and labeled. It can be observed that the T2638 site is invariant in all plants analyzed, indicating its importance for DNA-PKcs function across different species. Furthermore, T2609 site is conserved in most of the plant DNA-PKcs homologs examined, suggesting a possible functional role in these organisms.

Bp- *Bathycoccus prasinos*; Ot- *Ostreococcus tauri*; Me- *Mesotaenium endlicherianum*; Kn- *Klebsormidium nitens*; Mp- *Marchantia polymorpha*; Pp- *Physcomitrella patens*; Sf- *Sphagnum fallax*; Aa- *Anthoceros agrestis*; Dc- *Diphasiastrum complanatum*; Sm- *Selaginella moellendorffii*; Cr- *Ceratopteris richardii*; Af- *Azolla filiculoides*; Ss- *Sequoia sempervirens*; Pt- *Pinus taeda*; Hs-*Homo sapiens*.

Supplementary Figure 7.

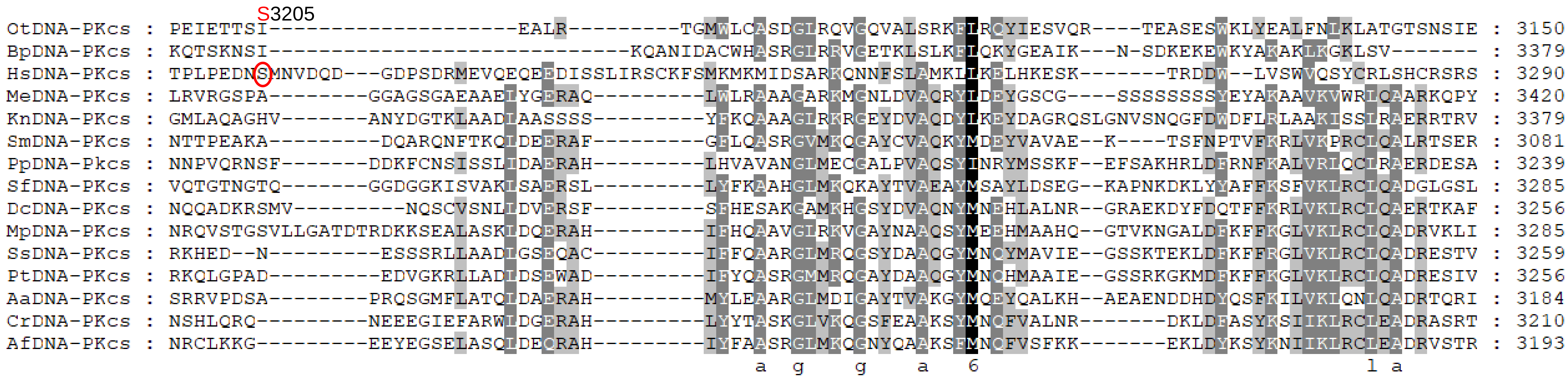

Supplementary Figure 7. Conservation of DNA-PKcs S3205 autophosphorylation site in plant lineages. Amino acid alignment of the FAT domain region of DNA-PKcs in selected plant species. The S3205 site in human DNA-PKcs is highlighted in red, and the lack of conservation in green plants can be observed.

Supplementary figure 8.

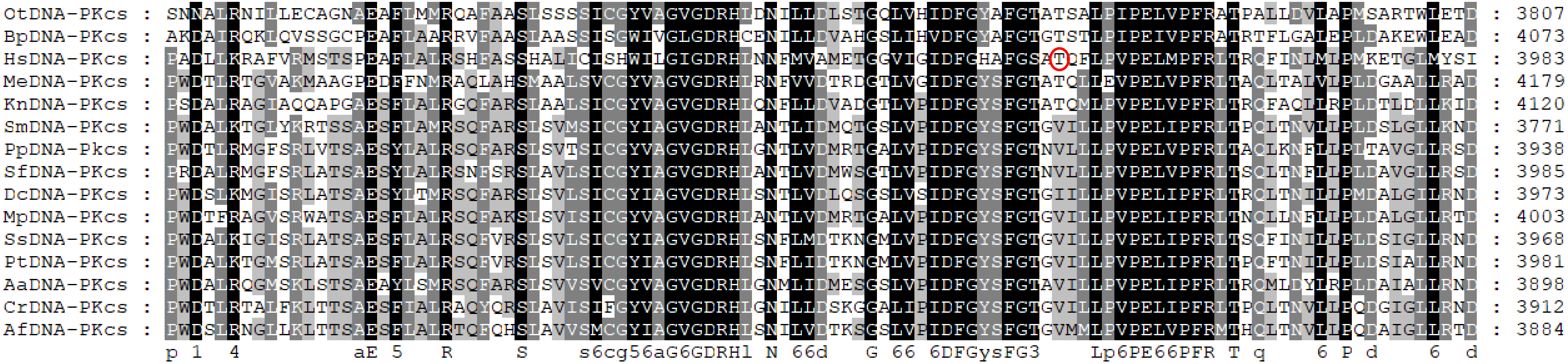

Supplementary Figure 8. Conservation of DNA-PKcs T3950 autophosphorylation site in plant lineages. Amino acid alignment of the kinase domain of DNA-PKcs from different plant lineages. The T3950 site is highlighted in red, and the substitutions by valine and isoleucine in some plant lineages can be observed.

Supplementary figure 9.

```
# -----------------
# LOCALIZER 1.0.4 Predictions
# -----------------
Identifier      Chloroplast             Mitochondria            Nucleus
BpDNA-PKcs      -                       -                       Y (KKRK,RASKRRI,RKYRALQKHSREQRK,KRRISSSGSDTKRRSQ,KRKDETATSLEARKYRA,KKNRVLFHTEVNHEKEKKE)
OtDNA-PKcs      -                       -                       Y (KRKR,KRSIVIWGGGKKRKQL)
MeDNA-PKcs      -                       -                       Y (KRRS,RRKR,RRRR,RKYLREVLERSRRYKQ)
KnDNA-PKcs      -                       -                       Y (KKPR,KPRK,KRAR,RPRR,RRRR,SKRQKGR,RKFLREVLVASRRYKL,KRNTADMTRTQARRQRE,RRGEELREEYQFQKIYRK,RRPVREGVAAAL[illegible]
MpDNA-PKcs      -                       -                       Y (KRQK,RKNEKDQSVYLDKIYKR)
PpDNA-Pkcs      -                       -                       Y (KKRK,KRAR,KRQK,RKYLQEVLVASRRYKL,RRRGEDSQSVHFDKIYKR,KKLQQLLVYIETERRKLSK)
SfDNA-PKcs      -                       -                       Y (KRAR,RKRK,RNKKRHR,RKPDMNNSDVKQKRL)
AaDNA-PKcs      -                       -                       Y (KRKH,RRYLQEVLVASRRYKL,KRVQHSSVVRASAIRGKRN)
DcDNA-PKcs      -                       -                       Y (RRTASRNSEINYERLYRK,KRLAKRNIDVLQKERKDRL)
SmDNA-PKcs      -                       -                       Y (KRHR,RKRK,RKKTMDSKTLKLLEKEKTK)
CrDNA-PKcs      -                       -                       Y (KRKL)
AfDNA-PKcs      -                       -                       Y (RRYLQEVLISSKRYKL,RKNMPWQYVGNVRKRHKG,RKHYYKILVYLYTERKMER)
SsDNA-PKcs      -                       -                       Y (LGEV,RKYLQEVLVASRRYKL,RRNITILLKNRKDRRM,RRKGQEHQSVHYEKIYRR)
PtDNA-PKcs      -                       -                       Y (KRKL,RRNISSLQENRKDRKM)



# Proteins analyzed: 14

# Number of proteins with cTP: 0 (0.0%)
# Number of proteins with cTP & possible mTP: 0 (0.0%)
# Number of proteins with cTP & NLS: 0 (0.0%)
# Number of proteins with cTP & possible mTP & NLS: 0 (0.0%)
# Number of proteins with mTP: 0 (0.0%)
# Number of proteins with mTP & possible cTP: 0 (0.0%)
# Number of proteins with mTP & NLS: 0 (0.0%)
# Number of proteins with mTP & possible cTP & NLS: 0 (0.0%)
# Number of proteins with NLS and no transit peptides: 14 (100.0%)

# Summary statistics

# Number of proteins with chloroplast localization (cTP, cTP & possible mTP, cTP & NLS, cTP & possible mTP & NLS): 0 (0.0%)
# Number of proteins with mitochondrial localization (mTP, mTP & possible cTP, mTP & NLS, mTP & possible cTP & NLS): 0 (0.0%)
# Number of proteins with nuclear localization and no transit peptides: 14 (100.0%)
# Number of proteins with nuclear localization and with transit peptides: 0 (0.0%)
```

Supplementary figure 9. Subcellular localization prediction of putative plant DNA-PKcs proteins. LOCALIZER (https://localizer.csiro.au/) was used to predict the subcellular localization. All the 14 proteins used in the current study are predicted to be localized to nucleus.

Supplementary figure 10.

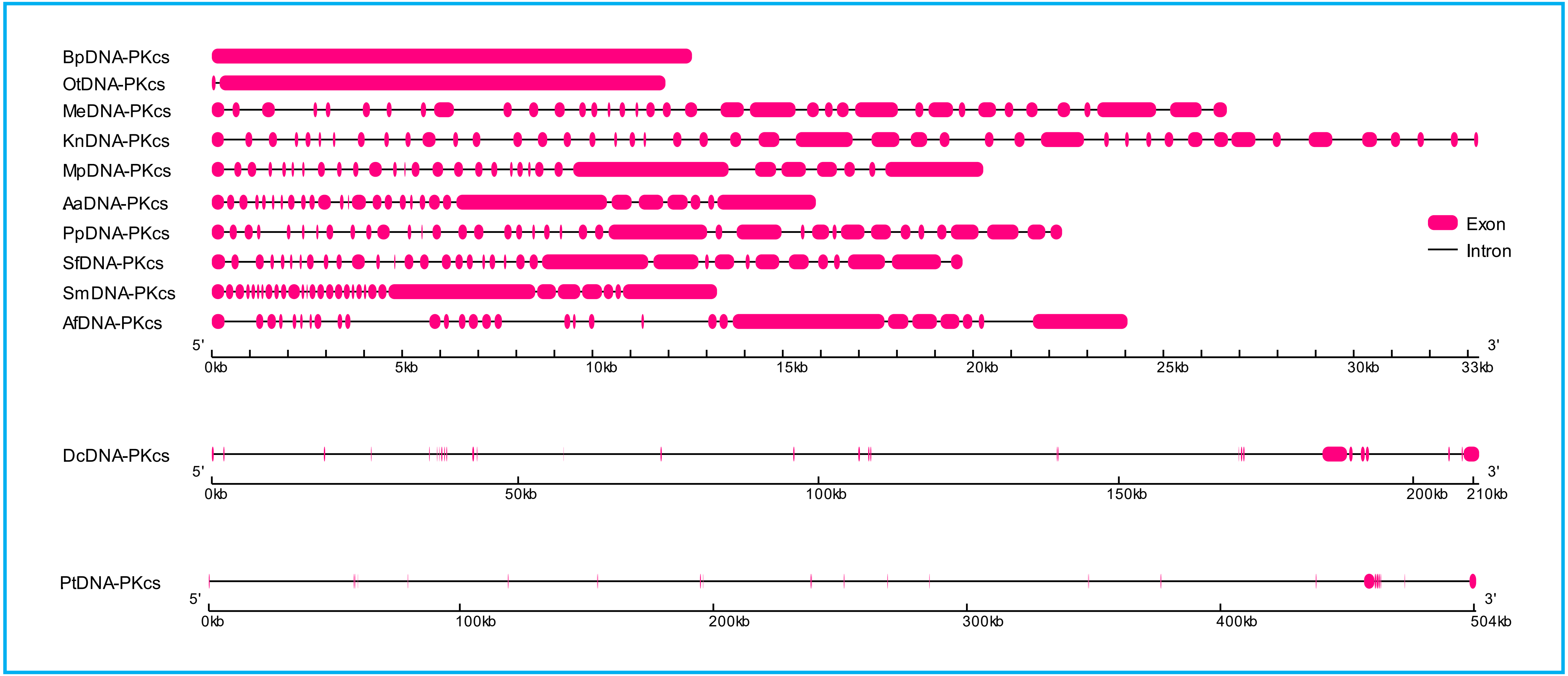

Supplementary Figure 10. Gene length and putative gene structure of DNA-PKcs from different plant lineages. Organisms with no complete gene sequence available, due to its representation in two different genome scaffolds or partially missing from genome scaffolds are excluded. Introns and exons are represented by dark lines and pink boxes, respectively.
