# Supplementary material for: Lost in the bloom: DNA-PKcs in green plants
Source: Front Plant Sci. 2023 Jul 28;14:1231678. doi: 10.3389/fpls.2023.1231678 (PMC10419180; doi:10.3389/fpls.2023.1231678)
Supplement: Supplementary Table 1 — Details of putative DNA-PKcs gene/protein annotations used or discussed in the current study from different plant species. [file DataSheet_1.zip › Supplementary Table.PDF]

**Organism**	**Gene/protein annotation discussed or used in this study**		**Annotation/prediction supported by transcriptome data of the organism or a closely related species (based on TSA available publicly)**	
	**Existing gene annotation/prediction: YES/NO (Details)**	**Existing gene annotation/prediction either modified /New annotation/prediction for this study: YES/NO (Details)**	**Partially supported: YES/NO (Details)**	**Completely supported: YES/NO (Details)**
Chlamydomonas eustigma	NO (GAX74142, GAX74143) NCBI	NO	YES (GFWA01012995.1; GFWA01107356.1) (*Chlamydomonas acidophila*, NCBI)	No
Volvox carteri	NO (Vocar.0009s0222) Phytozome	NO	NO	NO
Bathycoccus prasinos	YES (XP_007511896.1), NCBI	NO	YES (HBMR01008367; HBMR01008373; HBMR01008370), NCBI	NO
Ostreococcus tauri	YES (OUS42981.1), NCBI	NO	YES (HBDX01000727; HBLQ01004044), NCBI	NO
Mesotaenium endlicherianum	NO (ME000610S08690)	YES, (Described in methods)	NO	NO
Chara braunii	NO (GBG89877.1)	NO	YES (GGXX01117481.1), NCBI	NO

Klebsormidium nitens	NO (GAQ84758.1 , GAQ84757.1) Source: NCBI	YES (Described in methods)	YES (GBSO01008411.1) (GBSO01005714.1), NCBI	NO
Marchantia polymorpha	YES (Mapoly0069s0061.1) Source: Phytozome	NO	YES (GEFO01007190.1, GEFO01010847.1)	NO
Physcomitrella patens	YES (XP_024384633.1) Source: NCBI	NO	NO	YES (as per entry XM_024528865, 100% coverage of the annotated genomic feature by RNAseq alignments)
Sphagnum fallax	YES<br><br>(Sphfalx06G105000.1)<br><br>Source: "Sphagnum fallax v1.1, DOE-JGI,<br><br>http://phytozome.jgi.doe.gov/".	NO	NO	NO

Anthoceros agrestis [Oxford]	NO (AagrOXF_evm.model.utg000049l.749.1) Source: https://www.hornworts.uzh.ch	YES (Described in methods) (scaffold_2012162, 1KP)	NO	YES (scaffold_2012162), 1KP
Diphasiastrum complanatum	YES (Dicom.17G090200.1)<br><br>Source: "*Diphasiastrum complanatum* v3.1, DOE-JGI, http://phytozome-next.jgi.doe.gov/	NO	NO	NO
Selaginella moellendorffii	YES (XP_002965996.2)<br><br>XP_002989230.2	NO	NO	YES (NCBI entry:100% coverage of the annotated genomic feature by RNAseq alignments" And (GEMU01096076.1)
Azolla filiculoides	NO (Azfi_s0095.g043658)<br><br>Source: Fernbase.org	YES<br><br>(Described in methods)	YES (partial TSA, at NCBI) GBTV01032116.1 GBTV01032115.1 and at fernbase.org	
salvinia cucullata	NO (Sacu_v1.1_s0105.g020212) Source: Fernbase.org	NO	YES (at fernbase.org)	NO

Ceratopteris richardii	NO (Ceric.1Z120700)<br><br>Source: Phytozome	YES (based on TSA)	No	YES (GIXU01015881<br>)
Pinus taeda	NO	YES (Predicted from TSA, Described in methods)	Yes<br>*Pinus taeda* TSA (GIYS01087684)	YES (GHWB01310569)(*Pinus flexilis* TSA)
Sequoia sempervirens	NO	YES<br><br>Predicted from TSA (GIBU01226047)	No	YES (GIBU01226047)
Isoetes taiwanensis	NO<br>>Isoetes taiwanensis||Itaiw_v1_scaffold_24||14899984||14905902||Itaiw_v1_scaffold_24_t17467-RA||1||CDS||3473898239||3044||frame0<br>>Isoetes taiwanensis||Itaiw_v1_scaffold_24||14910580||14923530||Itaiw_v1_scaffold_24_t17468-RA||1||CDS||3473898242||4268||frame0	NO	NO	Annotated as two separate proteins.NO sufficient RNA support to determine the full length protein.

Supplementary Table 1. Details of putative DNA-PKcs gene/protein annotations used or discussed in the current study from different plant species.
